# Compliance with clinical practice guidelines for breast cancer treatment: a population-based study of quality-of-care indicators in Italy

**DOI:** 10.1186/1472-6963-13-28

**Published:** 2013-01-25

**Authors:** Carlotta Sacerdote, Rita Bordon, Sabina Pitarella, Maria Piera Mano, Ileana Baldi, Denise Casella, Daniela Di Cuonzo, Alfonso Frigerio, Luisella Milanesio, Franco Merletti, Eva Pagano, Fulvio Ricceri, Stefano Rosso, Nereo Segnan, Mariano Tomatis, Giovannino Ciccone, Paolo Vineis, Antonio Ponti

**Affiliations:** 1 Cancer Epidemiology Unit, San Giovanni Battista Hospital, CPO Piemonte and University of Turin, Turin, Italy; 2 Cancer Epidemiology Unit, San Giovanni Battista Hospital, CPO Piemonte, Turin, Italy; 3 Screening Centre, Department of Cancer Prevention, San Giovanni Battista Hospital, CPO Piemonte, Turin, Italy; 4 HuGeF Foundation, Via Nizza 52, 10126, Torino, Italy; 5 Department of Epidemiology and Biostatistics, School of Public Health, Imperial College, London, UK; 6 Cancer Epidemiology Unit, San Giovanni Battista Hospital, Via Santena 7, 10129, Torino, Italy

**Keywords:** Breast cancer, Guideline adherence, Population-based, Evidence-based medicine, Quality of care

## Abstract

**Background:**

It has been documented that variations exist in breast cancer treatment despite wide dissemination of clinical practice guidelines. The aim of this population-based study was to evaluate the impact of regional guidelines (Piedmont guidelines, PGL) for breast cancer diagnosis and treatment on quality-of-care indicators in the Northwestern Italian region of Piedmont.

**Methods:**

We included two samples of women aged 50–69 years with incident breast cancer treated in Piedmont before and after the introduction of PGL: 600 in 2002 (pre-PGL) and 621 in 2004 (post-PGL). Patients were randomly selected among all incident breast cancer cases identified through the hospital discharge records database. We extracted clinical data on breast cancer cases from medical charts and ascertained vital status through linkage with town offices. We assessed compliance with 14 quality-of-care indicators from PGL recommendations, before and after their introduction in clinical practice.

**Results:**

Among patients with invasive lesions, 77.1% (N = 368) and 77.5% (N = 383) in the pre-PGL and post-PGL groups, respectively, received breast conservative surgery (BCS) as a first-line treatment. Following BCS, 87.7% received radiotherapy in 2002, compared to 87.9% in 2004. Of all patients at medium-to-high risk of distant metastasis, 65.5% (N = 268) and 63.6% (N = 252) received chemotherapy in 2002 and in 2004, respectively. Among the 117 patients with invasive lesions and negative estrogen receptor status in 2002, hormonal therapy was prescribed in 23 of them (19.6%). The incorrect prescription of hormonal therapy decreased to 10.8% (N = 10) among the 92 estrogen receptor-negative patients in 2004 (p < 0.01).

Compliance with PGL recommendations was already high in the pre-PGL group, although some quality-of-care indicators did not reach the standard. In the pre/post analysis, 8 out of 14 quality-of-care indicators showed an improvement from 2002 to 2004, but only 4 out of 14 reached statistical significance. We did not find any change in the risk of mortality in the post-PGL *versus* the pre-PGL group (adjusted hazard ratio 0.94, 95%CI 0.56–1.56).

**Conclusions:**

These results highlight the need to continue to improve breast cancer care and to measure adherence to PGL.

## Background

Evidence-based guidelines serve as a tool to ensure that patients receive treatment based on the best available evidence. In 1995, Sainsbury et al. postulated that the improvement of clinical practice in breast cancer treatment could increase 5-year survival by up to 10% [1].

In the Piedmont Region (Northwestern Italy, population 4.25 million), clinical practice guidelines (PGL) for the treatment of breast cancer were first released in July 2002 and disseminated to all relevant clinicians and other stakeholders [2,3] Furthermore, as from 1996, the Piedmont Region has been covered by a breast screening program for all resident women aged 50–69 years.

It has been well documented that there is considerable variation in breast cancer treatment despite wide dissemination of clinical practice guidelines [4]. Variations are usually related not only to patient characteristics, such as age and educational level, but also to geographic area of residence and hospital and physician characteristics [5].

Although several studies have examined the different surgical and medical breast cancer treatments employed following the publication of clinical practice guidelines, to our knowledge few reports have included a comparison with clinical practice prior to guideline publication, and even fewer have examined the impact of guidelines in clinical practice at a population level [6,7].

To evaluate the real impact of PGL on breast cancer treatment in Piedmont, we collected data from the medical charts of women with breast cancer aged 50–69 years to assess compliance with 14 quality-of-care indicators, based on PGL recommendations, before and after the introduction of PGL in clinical practice. Furthermore, we explored the use of post-surgical medical treatment, including radiotherapy, chemotherapy and hormonal therapy in the two periods, according to FIGO stage, lymph node involvement and hormone receptor status. Finally, we compared the survival rates of patients treated before and after the introduction of PGL.

## Methods

### Population and data sources

**Figure 1:**
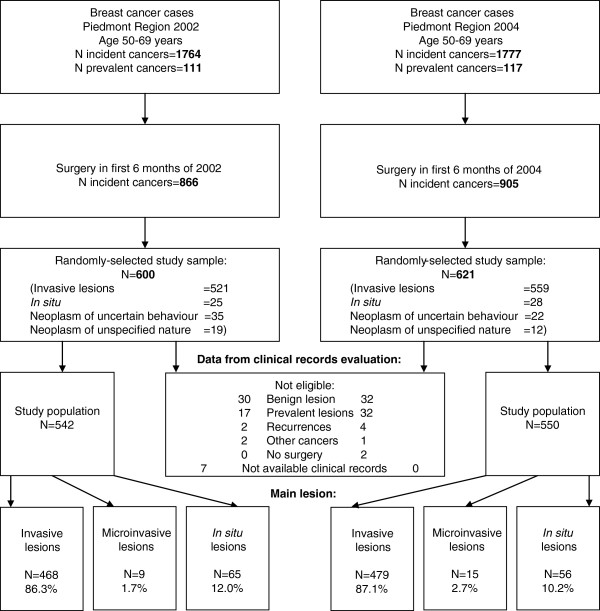
Study population of women with incident breast cancer who underwent surgery in 2002 and 2004.

The Piedmont hospital discharge records (HDR) database was used to identify female patients with incident breast cancer, aged 50–69 years, residing in Piedmont and surgically treated in 2002 and 2004 at regional hospitals. In 2002 we identified 1,764 female patients, of whom 866 underwent surgical treatment in the first 6 months of 2002. Among these 866 patients, 600 were randomly selected for this study (pre-PGL group). In 2004, 1,777 female patients were identified, of whom 905 underwent surgical treatment in the first 6 months of 2004. Among those 905 patients, 621 were randomly selected for this study (post-PGL group) (Figure 1). The start of the post-PGL period was 1.5 years after the introduction of PGL, as at this time they were likely to have been implemented in Piedmont hospitals.

We did not include prevalent breast cancer cases, i.e., women who had been previously hospitalized for breast cancer and were recorded in the Piedmont Cancer Registry and/or were in the HDR database between 1998 and 2002.

For the purposes of this study, breast cancer was defined by the following International Classification of Diseases 9^th^ Revision – Clinical Modification (ICD9-CM) codes: 174, carcinoma of the breast; 233.0, *in situ* carcinoma of the breast; 238.3, neoplasm of uncertain behavior of the breast; or 239.3, neoplasm of unspecified nature of the breast, in any position of the HDR database.

Fifty-eight patients from the randomly-selected pre-PGL group and 71 patients from the randomly-selected post-PGL group were not eligible for inclusion as they had benign lesions, prevalent lesions, recurrences, other cancers, no surgical treatment, or unavailable clinical records. After exclusion, 542 patients were left in the pre-PGL group and 550 in the post PGL-group, and were included in the following analyses (Figure 1).

*In situ* carcinomas of the breast were over-sampled in both groups. To do this, the same inclusion criteria were applied, but were not restricted to the first 6 months of 2002 and 2004. Instead, all women with *in situ* carcinoma (ICD9-CM code 233.0) who were surgically treated at any time in 2002 and 2004 were included. The total number of oversampled patients with *in situ* lesions was 121 in 2002 and 108 in 2004. This over-sampling was only used in the analyses concerning the indicators for patients with carcinoma *in situ* (CIS) in order to increase the power of the study.

The accuracy of the method adopted to identify incident breast cancer cases was validated using data from the Piedmont Cancer Registry, which covers about 20% of the regional population [8], as a gold standard. The sensitivity of the algorithm was 76.7% for breast cancer and the positive predictive value was 92.6%.

Follow-up of patients from both groups was performed through linkage to different databases using two hierarchical linkage keys. The HDR database was used to identify all subsequent relevant hospitalizations (2002–2005) for other surgical treatments (on the same, or other lesions), medical complications and chemotherapy sessions. Radiotherapy was assessed through linkage with the radiotherapy outpatient record database (which also includes extra-regional radiotherapy records), and hormonal therapy was assessed though linkage to the pharmaceutical prescription record database (which includes all drug prescriptions reimbursed to patients by the public health system).

All clinical records of any surgical or radiotherapy hospitalization identified for patients in the HDR database were retrieved. The clinical data were extracted from medical charts by two breast cancer screening technicians supervised by a gynecologist and an epidemiologist, working independently of the practitioners caring for patients in different hospitals. It was impossible to extract data blinded to the year of treatment since the data were obtained directly from the patients’ records. To control the extracted data, a random 10% sample was rechecked by the supervisors, blinded to the previous decisions. The collected data were entered into a database previously used for clinical audit (the Audit System on Quality of Breast Cancer Treatment), developed by a multidisciplinary team from the European Breast Cancer Network [9].

The ascertainment of vital status was carried out through linkage with town offices, identifying the date of death and allowing for the retrieval of the death certificate to identify the specific causes of death (2002–2010). All procedures concerning death certificates, data collection and coding were applied uniformly to both groups.

### Main outcome measures

Fourteen quality-of-care indicators were chosen to evaluate the impact of PGL (Table 1). We explored the use of the sentinel lymph node (SLN) technique by surgical unit annual case load, to measure the introduction of this procedure. Finally, we investigated post-surgical medical treatment, including chemotherapy among patients with invasive lesions at low/medium to high risk of distant metastasis (according to the Goldhirsch scheme) [10], and hormonal therapy prescribed according to estrogen receptor status.

**Table 1:** Piedmont Region clinical practice guidelines quality-of-care indicators

**Level of evidence (AHRQ grade of underlying recommendation)**	**Quality-of-care indicators**	**Function**	**Quality-of-care standards**
**B**	% of malignant lesions with cytological or histological pre-operative diagnosis	Calculates the proportion of patients with invasive or *in situ* lesions with a pre-operative cytological or histological diagnosis (C5 or B5), out of the total number of patients with invasive or *in situ* lesions who underwent surgical treatment.	**≥ 70%**
**A**	% BCS in pT1, unifocal	Calculates the proportion of patients diagnosed with invasive lesions of a pathological size of ≤20 mm (pT1, microinvasive included), not clinically multicentric or multifocal, who were treated with BCS.	**≥ 80%**
**B**	% BCS with free margins (>1 mm)	Calculates the proportion of BCS (last BCS if more than one) for invasive or *in situ *lesions which ensured clear margins (distance >1 mm from the lesion), out of the total number of BCS performed.	**/**
**/**	% single surgery after diagnosis	Calculates the proportion of patients whose first surgical treatment was not followed by further local operations that were required due to incomplete excision (excluding failed biopsies), out of the total number of patients who were surgically treated for localized invasive or *in situ *lesions with a positive or suspicious cytological or histological pre-operative diagnosis.	**/**
**C**	% frozen section in lesions ≤10 mm	Calculates the proportion of patients surgically treated for invasive lesions (excluding microinvasive lesions) of a maximum pathological size ≤10 mm for which there was no frozen section, out of the total number of patients with the same diagnosis.	**≥ 95%**
**A**	% of patients with invasive lesions treated with axillary clearance or SLN technique	Calculates the proportion of patients with invasive lesions who were treated with axillary dissection or SLN technique, out of the total number of patients with invasive lesions.	**/**
**C**	% of patients treated with axillary clearance with >9 lymph nodes	Calculates the proportion of patients with invasive lesions who were treated with axillary clearance (level I-III), excluding sampling, and from whom at least 10 lymph nodes were excised, out of the total number of patients with invasive lesions who were treated with axillary clearance.	**≥ 95%**
**C**	% with NO dissection among CIS patients	Calculates the proportion of patients diagnosed with CIS or not otherwise specified *in situ *lesions (microinvasive cancer excluded) on whom no axillary dissection was performed (not even level I) out of the total number of patients with this diagnosis who were surgically treated.	**≥ 95%**
**C**	% correct SLN identification	Calculates the% of SLN identified out of the total of identified SLN in patients with invasive lesions, who were treated with SLN technique.	**≥ 90%**
**C**	% histopathological grading available	Calculates the proportion of patients with invasive lesions (excluding microinvasive cancer) who were surgically treated and for whom measuring was provided, out of the total number of patients with invasive lesions who underwent surgical treatment.	**≥ 95%**
**C**	% hormonal receptor availability	Calculates the proportion of patients with invasive lesions (excluding microinvasive cancer) who were surgically treated and for whom measuring was provided, out of the total number of patients with invasive lesions who underwent surgical treatment.	**≥ 95%**
**/**	% immediate reconstruction after mastectomy	Calculates the proportion of patients with invasive or *in situ* lesions who had mastectomies and immediate reconstruction, out of the total number of patients treated with mastectomy.	**/**
**A**	% radiotherapy in patients treated with BCS	Calculates the proportion of patients who were treated with BCS for invasive or *in situ* lesions, and for whom radiotherapy followed, out of the total number of patients with the same diagnosis who were treated with BCS.	**≥ 95%**
**A**	% of eligible patients that receive hormonal therapy	Calculates the proportion of patients with invasive lesions and positive estrogen receptors who received hormonal therapy.	**/**

AHRQ grade of underlying recommendation (reference http://archive.ahrq.gov/clinic/epcarch.htm), BCS: breast conservative surgery, pT1: small primary tumor, SLN: sentinel lymph node, CIS: carcinoma *in situ.*

### Statistical analyses

The differences in distribution between post-PGL and pre-PGL breast cancer cases according to patient, tumor and surgical unit characteristics and in the rates of patients receiving post-surgical medical treatment were assessed by two-way Chi square Test or Fisher Exact Test.

The 14 quality-of-care indicators (Table 1) were analyzed as dichotomous variables by multivariable logistic regression models, using the period (post-PGL *versus* pre-PGL) as the main effect and controlling for confounders (age, educational level, clinical stage, screening provenience and surgical unit annual case load). The results are presented as frequencies, adjusted odds ratios (OR_adj_) and 95% confidence intervals (95%CI) estimated from logistic models to measure the probability of achieving standards in 2004 compared to 2002. We performed Cox proportional hazards regression analyses to study survival. Proportional hazard assumptions were tested with the Grambsch and Therneau test before analysis. Statistical analyses were performed using SAS 8.2 and STATA v10.

## Results

### Patient characteristics

Among the women included in the analyses, the distribution by type of lesion in the pre-PGL group was: 88.0% invasive or microinvasive (N = 477) (ICD9-CM code 174), and 12.0% *in situ* (N = 65) (ICD9-CM code 233.0); in the post-PGL group it was 89.8% invasive or microinvasive (N = 494), and 10.2% *in situ* (N = 56) (non-statistically significant difference) (Table 1).

Table 2 shows the distribution of breast cancer cases by patient, tumor and care provider characteristics for the pre-PGL and post-PGL groups. Both groups had similar age and educational level distribution. In contrast, there was a statistically significant difference in the distribution of clinical stages between 2002 and 2004. The proportion of cases diagnosed through the regional screening program increased from 38.4% (N = 208) in 2002 to 42.9% (N = 236) in 2004 (p = 0.001). Of all patients diagnosed with CIS, the percent diagnosed by the regional screening program increased from 45.9% (N = 28) in 2002, to 63.6% (N = 35) in 2004 (data not shown). The annual case load of surgical units was stable over the two periods (Table 2).

**Table 2:** Distribution of breast cancer cases according to various patient, tumor and surgical unit characteristics, 2002 and 2004, Piedmont Region, Italy

	**N and% of patients**		**Effect of year**
	**2002**	**2004**	**(p value)**
**Sample size (N)**	542 (49.6%)	550 (50.4%)	
**Age (years)**			
50–54	134 (24.7%)	124 (22.5%)	0.35
55–59	111 (20.5%)	122 (22.2%)	
60–64	166 (30.6%)	151 (27.5%)	
65–69	131 (24.2%)	153 (27.8%)	
Missing	0	0	
**Educational level**			
Bachelor	69 (12.7%)	99 (18.0%)	0.11
Secondary	151 (27.9%)	146 (26.5%)	
Professional	222 (40.9%)	197 (35.8%)	
Intermediate and primary	85 (15.7%)	88 (16.0%)	
None and unknown	15 (2.8%)	20 (3.6%)	
**Pathological T stage**			
*In Situ*	62 (11.4%)	55 (10.0%)	0.54
1mic	10 (1.8%)	15 (2.7%)	
1	4 (0.7%)	5 (0.9%)	
1a	17 (3.2%)	25 (4.5%)	
1b	75 (13.8%)	85 (15.5%)	
1c	186 (34.3%)	189 (34.4%)	
2	147 (27.2%)	136 (24.7%)	
3	5 (0.9%)	10 (1.8%)	
4	5 (0.9%)	5 (0.9%)	
4a	3 (0.6%)	0 (0%)	
4b	18 (3.3%)	15 (2.7%)	
4c	1 (0.2%)	0 (0%)	
4d	2 (0.4%)	2 (0.4%)	
X	4 (0.7%)	2 (0.4%)	
Missing	3 (0.6%)	6 (1.1%)	
**Pathological N stage**			
0	205 (43.3%)	158 (31.3%)	<0.01
Sentinel lymph node	80 (16.9%)	167 (33.1%)	
1	21 (4.4%)	37 (7.3%)	
1a	20 (4.2%)	72 (14.3%)	
1b	7 (1.5%)	3 (0.6%)	
1b1	40 (8.4%)	2 (0.4%)	
1b2	7 (1.5%)	3 (0.6%)	
1b3	56 (11.8%)	3 (0.6%)	
1b4	11 (2.3%)	3 (0.6%)	
2	15 (3.2%)	25 (5.0%)	
3	0	23 (4.6%)	
X	9 (1.9%)	6 (1.2%)	
Missing	2 (0.4%)	2 (0.4%)	
**Pathological TNM stage**			
*In situ*	65 (12.0%)	56 (10.2%)	0.04
I	189 (34.8%)	233 (42.3%)	
II A	127 (23.4%)	112 (20.4%)	
II B	75 (13.8%)	53 (9.6%)	
III or more	47 (8.8%)	65 (11.9%)	
Missing	39 (7.2%)	31 (5.6%)	
**Grading (invasive only)**			
Low	58 (12.0%)	82 (16.5%)	0.09
Intermediate	69 (14.4%)	86 (17.4%)	
High	103 (21.5%)	89 (18.4%)	
Not performed and missing	250	238	
**Disease detected through regional screening program**			
Yes	208 (38.4)	236 (42.9%)	0.001
No, symptomatic	209 (38.6%)	171 (31.9%)	
No, asymptomatic	110 (20.3%)	112 (20.4%)	
Missing	15 (2.8%)	31 (5.6%)	
**Surgical unit annual case load**			
< 50	124 (22.9%)	126 (22.9%)	0.001
50–149	249 (45.9%)	250 (45.5%)	
≥ 150	155 (28.6%)	147 (26.8%)	
Missing	14 (2.6%)	27 (4.8%)	

### Surgical and medical treatment

**Figure 2:**
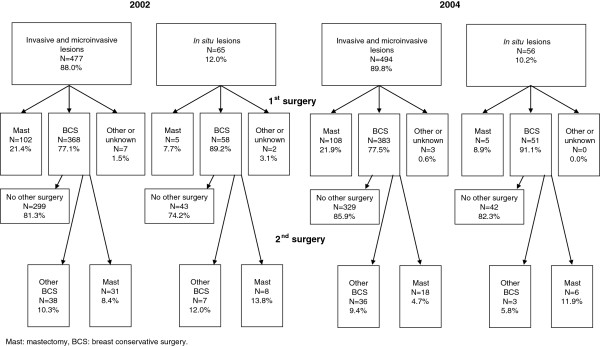
Surgical treatment in women with incident breast cancer who underwent surgery during the first 6 months of 2002 and 2004.

In the pre-PGL group 77.1% (N = 368) of patients with invasive lesions received BCS as a first-line treatment, and for 62.7% (N = 299) of patients in the pre-PGL group, BCS was a definitive surgical treatment. In the post-PGL group the numbers were 77.5% (N = 383) and 66.6% (N=329), respectively (Figure 2).

**Figure 3:**
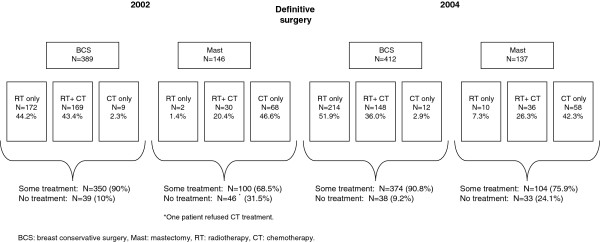
Post-surgical medical treatment in women with incident breast cancer who underwent surgery during the first 6 months of 2002 and 2004.

As for post-surgical medical treatment, following BCS, 87.7% (N = 341) of the pre-PGL group and 87.9% (N = 362) of the post-PGL group received radiotherapy alone, or in combination with chemotherapy (Figure 3). Post-surgical treatment with adjuvant chemotherapy was received by 50.9% (N = 276) of the pre-PGL group (about 46% following BCS and 67% following mastectomy) and by 46.2% (N = 254) of the post-PGL group (about 39% following BCS and 69% following mastectomy) (data not shown).

Post-surgical medical treatment of invasive lesions is shown in Tables 3 and 4. Of all patients with invasive lesions at medium-to-high risk of distant metastasis, 64.6% received chemotherapy (N = 520): 65.5% in the pre-PGL group and 63.6% in the post-PGL group. Among 113 women with invasive lesions at low risk of distant metastasis 14.3% and 1.4% received chemotherapy in 2002 and 2004, respectively (Table 3).

**Table 3:** Post-surgical chemotherapy by risk of distant metastasis according to ER and lymph node status, 2002 and 2004, Piedmont Region, Italy

	Low Risk*	**Medium-to-high risk§**	**p value**
	**N and %**	**N and %**	
**2002**	6/42	268/409	0.04
	(14.3%)	(65.5%)	
**2004**	1/71	252/396	
	(1.4%)	(63.6%)	

ER: estrogen receptor.

* ER-positive and size ≤ 2 cm and grading=1 with negative lymph node status.

§ ALL patients with positive lymph node status or estrogen receptor-negative or size > 2 cm or grading ≥ 2 regardless of lymph node status.

**Table 4:** Post-surgical hormonal therapy in patients with invasive lesions according to estrogen receptor (ER) status, 2002 and 2004, Piedmont Region, Italy

	**Estrogen receptor-positive**	**Estrogen receptor-negative**	**p value**
	**N and %**	**N and %**	
**2002**	319/338	23/117	< 0.01
	(88.0%)	(19.6%)	
**2004**	332/354	10/92	
	(89.8%)	(10.8%)	

Hormonal therapy was prescribed to 684 out of 901 (75.9%) patients with invasive lesions. Of women who were prescribed hormonal therapy, 4.8% (N = 33) had negative estrogen receptor status. The incorrect prescription of hormonal therapy in patients with negative receptor status decreased from 19.6% (N = 23) in 2002 to 10.8% (N = 10) in 2004 (p<0.01) (Table 4).

### Compliance with PGL recommendations

Univariate analysis showed that compliance with PGL recommendations was already high in the pre-PGL group. Indeed, five of nine quality-of-care indicators did not achieve the proposed standard (% of malignant lesions with cytological or histological pre-operative diagnosis, % frozen section in lesions ≤ 10 mm,% of patients treated with axillary clearance with > 9 lymph nodes, % with no dissection among CIS patients, % radiotherapy in patients treated with BCS).

In the pre/post analysis, studying the probability of achieving standards in 2004 compared to 2002, although eight of the 14 examined quality-of-care indicators changed in the expected direction, only four indicators improved substantially from 2002 to 2004: percent of malignant lesions with cytological or histological diagnosis before surgery (OR_adj_ 0.64, 95% CI 0.49–0.85), percent of BCS in pT1 lesions and percent of BCS performed with free margins (OR_adj_ 0.41, 95% CI 0.22–0.75 and OR_adj_ 0.65, 95% CI 0.41–1.01 respectively) and percentage of frozen section in lesions ≤ 10 mm (OR_adj_ 0.32, 95% CI 0.16–0.65). The other indicators were stable in the two periods (Table 5).

**Table 5:** Achievement of Piedmont Region clinical practice guidelines quality-of-care standards in 2002 and 2004, and effects of year, Piedmont Region, Italy

	**Results**				Effect of year (adjusted* OR and 95%CI) 2002 *vs *2004
	**2002**		**2004**		
	**N and %**	**Missing**	**N and %**	**Missing**	
% of malignant lesions with cytological or histological pre-operative diagnosis	297/513	48	329/495	65	0.64 (0.49-0.85)
	(57.9%)	(8.6%)	(66.5%)	(11.6%)	
% BCS in pT1, unifocal	231/268	0	253/272	0	0.41 (0.22-0.75)
	(86.2%)		(93.0%)		
% BCS with free margins (> 1 mm)	312/368	33	348/387	33	0.65 (0.41-1.01)
	(84.8%)	(8.2%)	(89.9%)	(7.9%)	
% single surgery after diagnosis	332/369	4	342/376	8	1.02 (0.24-5.91)
	(90.0%)	(1.1%)	(91.0%)	(2.1%)	
% frozen section in lesions ≤ 10 mm	55/97	1	85/113	0	0.32 (0.16-0.65)
	(56.7%)	(1.0%)	(75.2%)		
% of patients with invasive lesion treated with axillary clearance or SLN technique	433/468	0	460/478	1	0.28 (0.10-0.85)
	(92.5%)		(96.2%)	(0.2%)	
% of patients treated with axillary clearance with > 9 lymph nodes	317/336	4	245/268	4	1.71 (0.89-3.31)
	(94.3%)	(1.2%)	(91.4%)	(1.5%)	
% with NO dissection among CIS patients**	115/123	0	98/104	0	0.60 (0.14-2.50)
	(93.5)		(94.2%)		
% correct identification of SLN	118/126	30	215/219	54	0.46 (0.18-1.05)
	(93.6%)	(19.2%)	(98.2%)	(19.8%)	
% histopathological grading available	455/462	18	466/469	17	0.34 (0.08-1.42)
	(98.5%)	(3.7%)	(99.4%)	(3.5%)	
% hormonal receptor availability	455/471	9	446/473	13	1.80 (0.94-3.48)
	(96.6%)	(1.8%)	(94.3%)	(2.7%)	
% immediate reconstruction after mastectomy	41/154	4	30/137	2	1.16 (0.63-2.16)
	(26.6%)	(2.5%)	(21.9%)	(1.4%)	
% radiotherapy in patients treated with BCS	341/389	0	362/412	0	1.01 (0.60-1.5)
	(87.7%)		(87.9%)		
% of eligible patients that receive hormonal therapy	319/338	0	332/354	0	1.01 (0.69-1.42)
	(94.3%)		(93.8%)		

The 2004 period is the reference category for the estimation of ORs.

*Adjusted for age, educational level, clinical stage, screening provenience and surgical unit annual case load.

**Calculated on CIS lesions over-sampled group.

OR: odds ratio, BCS: breast conservative surgery, pT1: small primary tumor, CIS: carcinoma *in situ*, SLN: sentinel lymph node.

The number and percent of women treated with SLN technique by surgical unit annual case load is shown in Table 6. The PGL recommendations said that the SLN technique should be performed, as an alternative to axillary dissection, only by surgical units with a high annual case load. The percent of patients who were treated with SLN technique by a surgical unit with an annual case load of < 50 (based on the total number of women who underwent surgery in low-case load units) was 19.1% in 2002 (N = 25) and 42.3% in 2004 (N = 55). There was a big increase from 2002 to 2004 in the use of this technique across all strata of annual surgical case load, but we observed the biggest increase in the lowest category (annual case load < 50 = 55% increase, 50–149 = 40% increase, ≥ 150 = 41% increase) (Table 6).

**Table 6:** Surgical treatment with sentinel lymph node technique according to surgical unit annual case load, 2002 and 2004, Piedmont Region, Italy

**Surgical unit annual case load**	**Sentinel lymph node technique**			
	**2002**		**2004**	
	**N and %**	**Missing**	**N and %**	**Missing**
**< 50**	25/131	3	55/130	3
	(19.1%)	(2.2%)	(42.3%)	(2.2%)
**50–149**	67/244	7	113/250	1
	(27.4%)	(2.8%)	(45.2%)	(0.4%)
≥** 150**	55/146	8	95/149	0
	(37.7%)	(5.2%)	(63.7%)	
**Overall**	147/521	21	263/529	21
	(28.2%)	(3.9%)	(49.7%)	(3.8%)

### Survival analyses

Between 2002 and 2010, a total of 101 deaths were identified: 52 women in the pre-PGL and 49 in the post-PGL group (90.4% and 90.1% crude 5–year survival, respectively).

We did not find any change in the risk of mortality in the post-PGL *versus* the pre-PGL group (HR 0.94, 95%CI 0.56–1.56 adjusted for age, clinical stage and surgical unit annual case load).

## Discussion

This study evaluated compliance with PGL for breast cancer by comparing cases treated before and after their introduction. Patient and tumor characteristics were comparable between pre-PGL and post-PGL patients and the same methods of ascertainment and data collection were used.

In a previous report we investigated the distribution, implementation and evaluation of PGL among clinicians who treat breast cancer in the Piedmont Region. We found that approximately 90% of surgeons, gynecologists, oncologists and radiologists working in the field (70.2% of those who responded to the questionnaire), were aware of PGL within 1 year of their release, and generally had a positive attitude to change their practice accordingly [3].

In this population-based study we examined clinical practice patterns before and after the introduction of PGL. We and observed good compliance with PGL before their release, and a weak increase in the number of medical decisions that complied with them after their release.

The literature contains several examples of situations in which clinical practice guidelines on breast cancer treatment contributed to an improvement in quality of care [6,7,11,12], but very few of them included a comparison of clinical practice prior to the release of the guidelines [6], or examined this practice at a population level [7].

In 1997, Ray-Coquard and collaborators [6] conducted a study in France on 200 patients, with a before/after design, using information from medical records, and suggested significant changes in the quality of care. These changes were probably due to the introduction of clinical practice guidelines, but their results needed confirmation in a larger sample of cases.

White and collaborators [7] performed a study in Victoria, Australia in 2004 with a similar design using mailed questionnaires. All cases of early breast cancer registered in the Victorian Cancer Registry during two 6–month periods were selected and a questionnaire was sent to the relevant surgeon about patient characteristics and primary treatments. The study showed an improvement in quality of care after the introduction of clinical practice guidelines. However the study included data provided directly by surgeons from two different surveys. Significantly more surgeons completed the questionnaire in the first survey (73%) than in the second one (52%). The difference in the response rate between the two surveys could have introduced bias, causing a selection of those most interested in the topic, i.e., the surgeons with the highest case load [13]. Furthermore, answers furnished by the physicians could have reflected not necessarily what they *did* in their clinical practice, but what they knew they *should have done* to comply with the guidelines.

In our study we collected data directly from clinical records. We found a statistically significant positive trend in two of four quality-of-care indicators concerning diagnosis. In particular we found an improvement in the two indicators that were farthest from the standard in the pre-PGL group (% lesions with cytological/histological diagnosis before surgery and% frozen sections in ≤ 10 mm lesions), but not in the two indicators that already have a good compliance with PGL (percent of histopathological grading available and percent of hormone receptor available).

We measured two important quality-of-care indicators concerning the surgical treatment of breast cancer: percent of BCS in pT1 lesions and percent of BCS performed with free margins, and the results were positive. We noticed a trend of improvement in the post-PGL group: 93% of patients with pT1 were treated surgically with BCS in the present study. We found a similar positive trend concerning the practice of BCS over a 5–year period (2000–2004) in a previous population-based study on women with breast cancer (all ages) carried out in the Piedmont Region using administrative data [14]. Nevertheless, the percentage of single surgery after diagnosis, which was already good before PGL were released, and the percent of reconstruction after mastectomy, which was extremely low, did not show a positive trend over time.

The indicators regarding axillary surgery showed an increased proportion of patients that were treated with axillary clearance with a correct indication. The percent of patients with a clearance of > 9 lymph nodes and the percent of dissections not performed among CIS patients did not reach the standard and did not improve after the release of PGL. Conversely, a higher number of centers performed the SLN technique, with an identification rate that reached the standard.

Finally, looking at the proportion of women who underwent SLN technique by the annual case load of the surgical unit, we found that the use of this technique increased by almost 45% from 2002 to 2004, across all strata of surgical unit annual caseload but in particular in centers treating less than 50 breast cancer a year. This finding is clearly in contrast with the PGL recommendations that suggest the use of SLN technique only in specialized centers (surgical unit annual case load > 50). The increase of use of SLN technique in low caseload centers need to be discouraged.

Between 2002 and 2004 the proportion of women who received radiotherapy after breast cancer surgery (87.7% in 2002 and 87.9% in 2004) was stable, though still far from the standard of 95%. In a previous population-based study in Piedmont, the presence of a radiotherapy unit within the same hospital where the surgical procedure was performed was associated with a higher probability of receiving radiotherapy after discharge. The presence of a radiotherapy unit in the hospital also correlated with the case load and specialization of the surgical unit [14].

In the analyses of post-surgical medical treatment we found a decrease in the percent of patients with invasive lesions at medium-to-high risk of distant metastasis who received chemotherapy after the introduction of PGL. Furthermore, we observed a decrease in the percent of patients with invasive lesions and low risk of distant metastasis who received inappropriate chemotherapy. The percent of patients who received hormonal therapy was stable in the group with positive estrogen receptor status, and the incorrect prescription of hormonal treatment in estrogen receptor-negative women dramatically decreased in the post-PGL group.

Apart from the introduction of PGL, the positive trend in some of quality-of-care indicators can be partly attributed to the increased proportion of breast cancer cases diagnosed through the regional screening program. In fact, the patients who were diagnosed in the context of the screening program were usually referred to a surgical unit with a high annual case load.

Underestimation of chemotherapy, radiotherapy and/or hormonal therapy was possible given that these treatments are administrated at a different hospital admission than that for the surgical treatment, or even on an ambulatory basis. The information we collected about post-surgical medical treatment was the result of record-linkages between breast cancer patients and the HDR database, radiotherapy outpatient record database and pharmaceutical prescription record database. Such linkages can generate omissions that are likely to be random, so the resulting bias would be conservative.

Finally, we did not see changes in the survival rate between pre-PGL and post-PGL groups. In fact, the majority of PGL recommendations were oriented to avoid invasive surgery, over-treatment, recurrence, or patient anxiety (i.e., avoid mastectomy in pT1 unifocal, avoid more than one surgery after diagnosis, avoid dissection in CIS patients). Very few recommendations were formulated to improve survival (i.e., to measure hormonal receptor availability, to perform radiotherapy in patients treated with BCS, to perform axillary clearance or SLN technique in patients with invasive lesions). Moreover the indicators more related to survival, with the exception of axillary clearance, showed only a negligible improvement after the introduction of PGL. Finally it is possible that, since breast cancer is generally characterized by long survival, a small improvement in survival will manifest itself only with longer follow-up.

The population-based approach of the study ensures that selection bias was minimal and that the results can be considered representative of the entire Piedmont Region.

## Conclusions

The results suggest that the majority of quality-of-care indicators changed in the expected direction after the introduction of PGL, even if only 4 out of 14 reached statistical significance. Statistical significance was registered in particular in the indicators that were far from achieving the standard. Our results highlight the need to continue to improve breast cancer care and to measure the correct adherence to PGL.

In a previous study we evaluated the distribution, implementation and acceptance of PGL among medical doctors, and in the present paper we present the results based on quality-of-care indicators before and after the introduction of PGL. The ultimate evaluation must involve long-term outcome studies on recurrence and mortality to assess the real impact of PGL on patient care, and economic evaluations to assess treatment choices.

## Abbreviations

PGL: Piedmont guidelines; HDR: Hospital discharge records; N: Number; BCS: Breast conservative surgery; CIS: Carcinoma *in situ*; OR_adj_: Adjusted OR; CI: Confidence interval; SLN: Sentinel lymph node.

## Competing interests

The authors declare that they have no competing interests in connection with this paper.

## Authors’ contribution

CS and AP conceived of the study and co-wrote the manuscript with contributions from all other authors; RB and MPM contributed to study design and coordination of the study; SP, DC, RR, ST, AF and LM contributed to acquisition of the data and their interpretation; IB, EP, RR, DDC, MT and FR performed the statistical analysis and contributed to data interpretation; AP, GC, NS, FM, and PV conceived of the study, contributed to data interpretation, obtaining grant funding and financial support. All authors have given final approval of the version to be published.

## Pre-publication history

The pre-publication history for this paper can be accessed here:

http://www.biomedcentral.com/1472-6963/13/28/prepub
